# Macroepidemiological trends of Influenza A virus detection through reverse transcription real-time polymerase chain reaction (RT-rtPCR) in porcine samples in the United States over the last 20 years

**DOI:** 10.3389/fvets.2025.1572237

**Published:** 2025-04-24

**Authors:** Daniel C. A. Moraes, Guilherme A. Cezar, Edison S. Magalhães, Rafael R. Nicolino, Kinath Rupasinghe, Srijita Chandra, Gustavo S. Silva, Marcelo N. Almeida, Bret Crim, Eric R. Burrough, Phillip C. Gauger, Darin Madson, Joseph Thomas, Michael A. Zeller, Rodger Main, Mary Thurn, Paulo Lages, Cezar A. Corzo, Mattew Sturos, Hemant Naikare, Rob McGaughey, Franco Matias Ferreyra, Jamie Retallick, Jordan Gebhardt, Sara McReynolds, Jon Greseth, Darren Kersey, Travis Clement, Angela Pillatzki, Jane Christopher-Hennings, Beth S. Thompson, Melanie Prarat, Dennis Summers, Craig Bowen, Joseph Boyle, Kenitra Hendrix, James Lyons, Kelli Werling, Andreia G. Arruda, Mark Schwartz, Paul Yeske, Deborah Murray, Brigitte Mason, Peter Schneider, Samuel Copeland, Luc Dufresne, Daniel Boykin, Corrine Fruge, William Hollis, Rebecca C. Robbins, Thomas Petznick, Kurt Kuecker, Lauren Glowzenski, Megan Niederwerder, Daniel C. L. Linhares, Giovani Trevisan

**Affiliations:** ^1^Veterinary Diagnostic and Production Animal Medicine, Iowa State University, Ames, IA, United States; ^2^Veterinary Population Medicine, University of Minnesota, Saint Paul, MN, United States; ^3^Kansas State Veterinary Diagnostic Laboratory, Kansas State University, Manhattan, KS, United States; ^4^Kansas Department of Agriculture, Division of Animal Health, Manhattan, KS, United States; ^5^Veterinary and Biomedical Sciences Department, South Dakota State University, Brookings, SD, United States; ^6^South Dakota Animal Industry Board, Pierre, SD, United States; ^7^Ohio Animal Disease and Diagnostic Laboratory, Reynoldsburg, OH, United States; ^8^College of Veterinary Medicine, Purdue University, West Lafayette, IN, United States; ^9^Indiana State Board of Animal Health, Indianapolis, IN, United States; ^10^Department of Veterinary Preventive Medicine, College of Veterinary Medicine, The Ohio State University, Columbus, OH, United States; ^11^Schwartz Farms Inc., Sleepy Eye, MN, United States; ^12^Swine Vet Center, St. Peter, MN, United States; ^13^New Fashion Pork, Jackson, MN, United States; ^14^Country View Family Farms, Middletown, PA, United States; ^15^Innovative Agriculture Solutions, LLC, Waterloo, IA, United States; ^16^Prestage Farms, Clinton, NC, United States; ^17^Swine Veterinary Partners, Québec, QC, Canada; ^18^Smithfield Foods, Smithfield, VA, United States; ^19^The Maschhoffs LLC, Carlyle, IL, United States; ^20^Carthage Veterinary Service LTD, Carthage, IL, United States; ^21^Pig Improvement Company, Hendersonville, TN, United States; ^22^ArkCare, Omaha, NE, United States; ^23^The Hanor Company, Enid, OK, United States; ^24^Pipestone Veterinary Services, Pipestone, MN, United States; ^25^Swine Health Information Center, Ames, IA, United States

**Keywords:** zoonotic disease, IAV, monitoring, swine, epidemiology, diagnostic, forecasting

## Abstract

Influenza A virus (IAV) in swine is a major respiratory pathogen with global significance. This study aimed to characterize the macroepidemiological patterns of IAV detection using reverse transcription real-time polymerase chain reaction (RT-rtPCR) assays, including subtype identification, in samples submitted between January 2004 and December 2024 to veterinary diagnostic laboratories (VDLs) participating in the Swine Disease Reporting System (SDRS). A secondary objective was establishing an IAV monitoring capability to inform stakeholders of weekly changes in IAV detection patterns. Of the 372,659 samples submitted, 31% tested positive for IAV RNA via RT-rtPCR. The most frequent sample types were oral fluids (44.1%) and lung tissue (38.7%). Submissions from the wean-to-market category had a higher positivity rate (34.4%) than those from the adult/sow farm category (26.9%). IAV detection followed a seasonal pattern, with peaks in spring and fall and lower positivity rates in summer. Of the total of 118,490 samples tested for IAV subtyping using RT-rtPCR, the most frequently detected subtypes were H1N1 (33.1%), H3N2 (25.5%), H1N2 (24.3%), H3N1 (0.2%), mixed subtypes (5.4%), and partial subtype detection (11.5%). Mixed IAV subtypes were detected in individual samples—including lung tissue, nasal swabs, and bronchoalveolar lavage—indicating co-infection with two or more IAV strains. For IAV forecasting, a combined model using dynamic regression and a neural network outperformed individual models in 2023, achieving the lowest root mean square error (RMSE) and an improved overall skill score. This study highlights the importance of using laboratory submission data for IAV surveillance and macroepidemiological analysis. The findings provide valuable insights into IAV dynamics and highlight the need for standardized monitoring systems in VDLs to enhance understanding of IAV in swine populations across the United States.

## 1 Introduction

Influenza A virus (IAV) is classified within the *Orthomyxoviridae* family, consisting of enveloped virions with segmented, negative-sense ribonucleic acid (RNA) genomes (1). IAV is a significant respiratory pathogen affecting animal health worldwide, impacting various species, including swine and poultry (2), and it has zoonotic potential (3). Human IAV strains can be transmitted to swine and have significantly contributed to the genetic diversity of IAV circulating in swine populations globally—the most recent example being the reverse zoonoses of the 2022–2023 human seasonal H3N2 virus to swine (4, 5). These human seasonal influenza viruses influence the diversity of IAV in swine, complicating control efforts and leading to frequent incursions of human strains into swine populations (6). For example, the 2009 H1N1 influenza pandemic resulted in substantial economic losses for the US pork industry, estimated at over $1 billion, and created public misperceptions about pork safety (7). Recently, outbreaks of the highly pathogenic avian influenza A (HPAI) H5N1 virus in dairy cattle and cats, along with the detection of spillover events across a broad host range, are concerning and suggest an increasing potential for the virus to adapt to mammals, including livestock, pets, and humans (8, 9). Moreover, a recent study showed that pigs are susceptible to H5N1 infection and highlighted the importance of biosecurity in swine herds to protect against this virus incursion (10, 11). The first field case of H5N1 in swine was recently detected on a multi-species backyard farm in the United States (12).

Diverse IAV subtypes are classified by the hemagglutinin (HA) and neuraminidase (NA) glycoproteins that protrude from the surface of the viral envelope (13). The predominant subtypes in swine populations arise from various combinations of HA and NA, particularly H1 and H3, along with N1 and N2 (e.g., H1N1, H1N2, and H3N2), exhibiting substantial genetic diversity circulating globally (3, 13, 14). Although these subtypes have been detected more frequently, isolated occurrences of subtypes, such as H2N3 and H4N6, have been reported in North American swine populations (15, 16). These sporadic IAV subtype detections have been limited to circulation among the pig populations in which they were found and have not been observed outside those isolated cases or become endemic (17). The ongoing evolution of antigenic characteristics within IAV subtypes poses a significant challenge to animal health, with varying implications across different geographical regions (18, 19).

Beyond genetic diversity, IAV infections in swine may occur in combination with other pathogens, further complicating disease management. Between 2010 and 2019, 12,547 confirmed cases of IAV infection were reported to cause lesions in the respiratory tissues of pigs (20). Among these, IAV was co-diagnosed with porcine reproductive and respiratory syndrome virus (PRRSV) in 1,626 cases, representing 9.3% of all evaluated tissue samples. Co-infections with bacterial pathogens included 331 cases (2.1%) with *Streptococcus suis*, 322 (1.8%) with *Pasteurella multocida*, 256 (1.5%) with *Glasserella parasuis*, and 231 (1.3%) with *Mycoplasma hyopneumoniae* (20).

The dynamics and diversity of IAV require ongoing surveillance to enhance understanding and generate solutions for its control. A notable example is the United States Department of Agriculture (USDA) IAV Swine Surveillance program, which provides valuable insights into IAV ecology with data that may help identify influenza trends in swine. These data are made available to producers, swine practitioners, and diagnosticians (21). Although this program offers updates on national IAV swine surveillance, its voluntary nature limits its representation of the entire domestic swine population. Another monitoring tool is the ISU FLUture platform, which offers multiple web-based tools for analyzing IAV sequencing and case metadata from swine samples tested at the Iowa State University Veterinary Diagnostic Laboratory (ISU VDL) (22). FLUture helps further explore IAV clade trends and includes additional tools, such as the HA sequence identity tool, to determine the clade of a given sequence (22). However, this data is limited to submissions to the ISU VDL, restricting the broader understanding of macroepidemiological aspects of IAV activity in the United States (US) swine population. In addition, these tools primarily focus on retrospective analyses, and the lack of real-time forecasting capabilities in current systems limits the ability to predict and respond to deviations in IAV detection patterns, ultimately hindering efforts to guide control strategies and inform stakeholders proactively.

Testing using RT-rtPCR assays is widely employed by VDLs for IAV RNA detection and IAV subtyping in swine samples. RT-rtPCR diagnostic testing is one of the most commonly used molecular methods for confirming influenza virus genetic material detection (23) due to the fast and accurate method for detecting viral RNA (24), and is an important component of national surveillance programs, including the USDA IAV swine surveillance program (21). Several sample types are frequently tested for IAV, such as oral fluids, nasal swabs, and lung tissues (25, 26). New antemortem and less invasive sample types, including udder wipes and nasal wipes, are being developed and assessed for their effectiveness in IAV testing (27, 28) to improve detection capabilities. Additionally, recent studies have shown that pooling strategies can effectively detect IAV, with findings from research involving pooled udder wipes and nasal swabs conducted for IAV surveillance purposes (29, 30). Data from samples submitted for testing at VDLs, along with associated IAV testing results, provides an opportunity to better inform stakeholders about IAV detection and activity patterns. Although the US has a National Animal Health Laboratory Network (NAHLN), information on megatrends for IAV PCR-based detection from porcine samples tested within this network of laboratories is currently unavailable in the US.

An organized information hub, such as those available for PRRSV, enteric coronavirus, and porcine circovirus 2 and 3 (31–33), would enable a better understanding of detection and diversity for IAV, thus improving strategic surveillance and control of IAV by providing information that can be further used to assist veterinarians, pig producers, practitioners, and researchers in the decision-making process. Consequently, this work aimed to characterize the macroepidemiological aspects of IAV and its subtypes detected by RT-rtPCR assays from porcine samples over time and across age categories. A secondary aim was to determine the seasonal patterns of IAV detection and establish an IAV monitoring capability to inform stakeholders of weekly changes in IAV detection patterns and any deviations from expected levels.

## 2 Materials and methods

### 2.1 Data source

Data encompassing IAV RT-rtPCR and IAV RT-rtPCR subtyping from samples tested between January 2004 and December 2024 were sourced from six VDLs: the ISU VDL, the University of Minnesota VDL (UMN VDL), Kansas State VDL (KS VDL), South Dakota State University Animal Disease Research and Diagnostic Laboratory (SDSU ADRDL), the Ohio Animal Disease Diagnostic Laboratory (Ohio ADDL), and Purdue University Animal Disease Diagnostic Laboratory (ADDL), hereafter referred to as VDLs. Together, these participating laboratories account for over 97% of all porcine cases tested in the NAHLN laboratory network. Submission case metadata, including receipt date, site state, test, and testing results, were retrieved following previously established methodologies (31, 32). The receipt date was the submission time recorded by each VDL, while the site state refers to the geographic location based on the state information included in the submission. Briefly, retrospective historical data were retrieved from each VDL's Laboratory Information Management System (LIMS) and shared in a comma-separated value (CSV) file format. In addition, prospective data were made available to this project via CSV files retrieved through application programming interface (API) calls for daily updates or through messages sent to an HL7 database (https://vdl.iastate.edu/sdrs/Search), managed by the Swine Disease Reporting System (SDRS, https://www.fieldepi.org/SDRS). The shared data did not include ant VDL client identification (e.g., producer and veterinarian information).

### 2.2 Dataset organization

The dataset comprised several data variables such as the date, geographic region (state), RT-rtPCR test results, age category, and specimen type. Specimen information was organized using SNOMED CT terminology (https://www.snomed.org/value-of-snomedct). The Logical Observation Identifiers Names and Codes (LOINC; https://search.loinc.org/searchLOINC) serve as a universal coding system that standardizes data related to laboratory test procedures and results, and it was used for data collation. Submissions marked as research testing on the VDL submission forms were excluded from this study to ensure the analysis focused on IAV detection under field conditions.

Unlike RT-rtPCR testing for IAV detection, the data regarding the detection of specific IAV RT-rtPCR subtypes, namely H1, H3, N1, and N2, were collected and organized at the sample level, utilizing the same set of variables with the addition of a sample number identifier. The IAV subtypes, such as H1N1, H1N2, H3N2, and H3N1, were identified within the datasets shared by participant VDLs. Samples, where only hemagglutinin (e.g., H1, H3, or H1H3) or only neuraminidase (e.g., N1, N2, or N1N2) was detected, were labeled as “*partial*” detection. Conversely, samples with complete subtype detection (hemagglutinin and neuraminidase) along with one or more different hemagglutinin or neuraminidase subtypes, such as H1H3N1, were labeled as “*mixed*” detection.

To streamline the data organization process, data processing and collation utilized a web-based application developed in C# 10 (34), supported by the.NET 6 web framework (35). The data from each participant's VDL IAV RT-rtPCR were organized at the submission level and categorized according to the IAV RT-rtPCR subtype sample, collating it into an inter-VDL standardized format. Each VDL reported IAV sample testing results as positive, suspect, inconclusive, or negative. A submission was considered positive when at least one sample within it had a result reported as positive. A submission was classified as suspect when a sample was labeled as suspect, but no positive samples were present, even though a suspect result was reported by the participant VDLs. Moreover, an inconclusive result was recognized when there was a report of an inconclusive test result without any positive or suspect results in the submission. Submissions that reported test results as neither positive, suspect, nor inconclusive were classified as negative.

The retained variables were defined according to a previously implemented methodology for PRRSV, enteric coronavirus, and porcine circovirus under the SDRS project (31, 33, 36) and were used as follows:

1. Age category: This variable was divided into two phases: adult/sow farm and wean-to-market. Information for the age category was generated using a combination of the provided farm type and the ages of animals mentioned in the submission forms. The adult/sow farm phase comprised samples identified as collected from adults, boar studs, breeding herds, replacement gilts, and suckling piglets. The wean-to-market phase aggregated data included cases classified as nursery or grow-finish. Cases with an unspecified age category, non-animal, or environmental samples in the VDL submission forms were labeled as “unknown.” To assess the difference in the proportion of adult/sow farm and wean-to-finish phases, we conducted a chi-squared test using R Studio (37). The significance level was set at a *P*-value ≤ 0.05 to identify differences in IAV positivity.
2. Season: This variable represented the seasons of the year and was organized as follows: samples received in December, January, and February were categorized as Winter; March, April, and May as Spring; June, July, and August as Summer; and September, October, and November as Fall.
3. Specimen: This variable represents the sample fraction used for testing the submitted sample type at the VDLs. When more than one sample type is submitted for testing and analyzed for IAV in the same case, the specimen is labeled as “multiple” (e.g., when oral fluids and nasal swabs are included in the same submission case and tested for IAV).
4. When variable fields were not explicitly provided during the submission process or captured in the LIMS system, they were labeled as “Unknown.” Examples of variables where this mapping occurred include site state, specimen, and age category when information was unreported.

### 2.3 Data visualization

The collated VDL-anonymized aggregated data was stored in the SDRS server database at the ISU College of Veterinary Medicine. The data was permissioned and connected to a commercially available data visualization tool, Microsoft Power BI (Power Business Intelligence; Microsoft Corporation, Redmond, WA), enabling the information to be displayed and visualized in various settings with interactive charts and graphs. Power BI has a built-in tool for creating web-embedded links that display generated charts and graphs in online interactive dashboards with predefined filters: result, site state of the specimen, specimen, age category, year, and month.

### 2.4 Influenza A virus seasonality assessment

The seasonal pattern of IAV detection by RT-rtPCR was statistically tested using the Average Seasonal Index (ASI), a widely used measure in time series analysis that is particularly helpful for examining irregular and long-period dynamic series. The ASI measurement was adapted from an established methodology (38) to identify the seasonal dynamics present in weekly IAV detection. Its objective was to highlight seasonal patterns of IAV and provide an average view of a time series' behavior throughout different periods of the year, allowing for the assessment of IAV seasonality on a weekly basis. This study calculated ASI by dividing each week by a 52 week moving average using the *forecast package* (39). Subsequently, a moving average analysis was performed over a 52-week period, a method for smoothing time series and highlighting long-term patterns, using R Studio software (37).

### 2.5 Influenza A virus RT-rtPCR detection monitoring

The trends in IAV RNA detection were assessed using historical data to predict and anticipate the proportion of positive submissions over the next 52 weeks. This assessment aimed to outline the bi-cyclic and complex pattern of IAV detection, facilitating the identification of weeks with notable increases or decreases in the detection rate.

Initially, the IAV detection database results were organized by weekly counts of positive submissions, negative submissions, and total submissions tested. Then, the percentage of IAV-positive submissions was calculated each week by dividing the number of positive submissions tested for that week by the total number of submissions for that week. A total of 260 weeks were used to train and test the model, with the data divided into 80% for training. the four previous years (208 observation weeks) and 20% for testing and validation. A total of 208 observations from the four prior years of data, consisting of 52 or 53 weeks each year, were used for training, while 52.18 observations (weeks) were allocated for the test data (40, 41).

Five distinct predictive models were employed to capture the complex temporal dynamics of IAV detection, which exhibits seasonality, to forecast the predicted weekly levels of IAV detection for an upcoming year. The time series models included (1) a Seasonal Autoregressive Integrated Moving Average (SARIMA) (42), (2) a cyclic regression model (43), (3) a dynamic regression model (44), (4) a neural network, a machine learning model (45), and (5) the Prophet model, a Bayesian model (46). The performance of each of these forecasting models was then analyzed using the *tsibble* package (47) and fable package (48) in R Studio software (37), using a formula for each model developed in a previous methodology (49), which was briefly summarized.

Given the seasonal component of IAV in the swine population, a Seasonal Autoregressive Integrated Moving Average (SARIMA) was used to assess the non-seasonal part of the model (p, d, q) and the seasonal part of the model (P, D, Q) (42). Considering the IAV bi-cyclic pattern, a cyclic regression model was implemented to fit linear models to time series data, incorporating trend, and seasonality components. The TSLM approach was used to fit linear models to the time series data, including trend, and seasonality components. Fourier terms were employed to accommodate a long-term dataset comprising weekly data (43). The dynamic regression model forecasts time series data (50) using dynamic regression while incorporating Fourier terms to predict weekly trends. The Fourier terms (K = Ki, where *i* = 1 to 12) were used to capture seasonal patterns over an extended period of weekly data, while short-term time series dynamics were managed through ARIMA error modeling. Thus, Fourier terms were tested from K = 1 to K = 12 in the training data to determine the K with the lowest Akaike Information Criterion (AIC) (44). A neural network model can be applied to time series data by building a non-linear autoregressive model and is often employed to estimate non-linear mapping due to its potential for learning the underlying non-linear relationships between future outcomes and individual forecasts (51). The fifth model employed was the Prophet model, applicable to time series data that exhibit seasonality and multiple seasons of historical data (46). In addition, by default, in the *fable* package, order 10 was used for annual seasonality, and order 3 was used for weekly seasonality (44).

The model fit evaluated the autocorrelation of residuals using the Ljung-Box test in five distinct predictive models (52). This test assessed the null hypothesis that the residuals were not significantly correlated, and the null hypothesis was rejected if the *p-value* < 0.05, indicating significant autocorrelation (53). Additionally, a logarithmic transformation was applied to the time series data to address its non-stationarity by stabilizing the mean and variance, which were observed to vary over time (54).

After conducting the forecasts, the performance of the models was assessed using forecast accuracy measures, such as RMSE, Mean Absolute Error (MAE), and Mean Absolute Percentage Error (MAPE). These metrics were essential for evaluating forecasting accuracy and providing insights into the models' predictive capabilities (55). RMSE and MAE, being scale-dependent measures, are commonly used to assess forecast errors. Moreover, MAPE, a percentage error metric, facilitates the comparison of forecast performances across different datasets (39).

A combination approach was also used to enhance the robustness of the forecasting process. Following the previously described combination methodology (49), a combined model was structured by selecting the two models that exhibited superior performance after model fitting and forecasting. These were characterized by lower RMSE, MAE, and MAPE on the testing data. In the model selection process, smaller values indicate better accuracy, and the optimal forecasting model was chosen by comparing the outcomes of the analyses to identify the model with the lowest RMSE, MAE, and MAPE (56). Finally, a skill score evaluates model performance by assessing forecast accuracy relative to a benchmark, enabling comparisons across different methods (57), and a higher skill score indicates better model performance (57). The forecast combination reduces the risk of over-reliance on any single model, thereby improving the overall reliability of the forecast and preventing a single model from overfitting the training data. Additionally, the data from the combined model was visualized using the *ggplot2* package in (58) R Studio to describe the forecast in terms of weekly IAV percentual positive submissions. Prospective IAV RT-rtPCR detection was compared with predicted values to assess deviations from a 95% prediction interval of the predicted baseline values and to monitor inconsistencies with historically expected levels.

## 3 Results

The IAV datasets were collated from the six participant VDLs and effectively structured, connected, and displayed in a dashboard. In addition, the data was organized in an anonymized format, and the generated dashboards for IAV RT-rtPCR detection and Influenza A virus subtyping are openly available on the SDRS project website https://www.fieldepi.org/SDRS, at the SDRS Dashboard, under the PCR Dashboard for all analytes, selecting IAV, and under the Influenza A virus subtyping dashboard, selecting IAV subtyping.

### 3.1 IAV RT-rtPCR results

**Figure 1:**
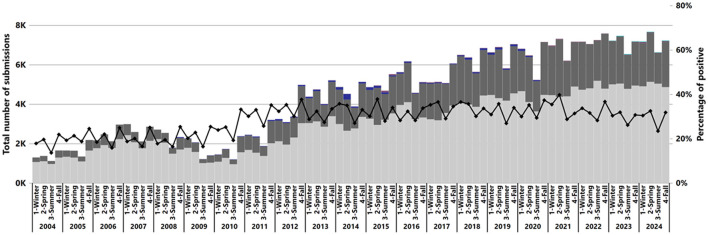
Number of submissions by result and percentage of positive submissions from the total IAV submissions tested by RT-rtPCR from 2004 to 2024. Each year is represented by the four seasons: 1, Winter; 2, Spring; 3, Summer; 4, Fall. The bars show the number of cases tested, with colors indicating test results: dark gray for positive, light gray for negative, blue for suspect, and purple for inconclusive (x-axis). A black line represents the percentage of positive cases on the secondary y-axis.

The number of IAV submissions increased from 5,622 cases in 2004 to 28,256 cases in 2024 (Figure 1), with the average number of submissions per month increasing from 469 cases in 2004 to 2,355 cases in 2024. Of the 372,659 total submissions tested, 31% of submissions (113,952) contained at least one RT-rtPCR-positive sample for IAV RNA. The number of positive submissions increased over time, with the annual average positivity rate rising from 19% (1,067 of 5,622) in 2004 to 29% (8,262 of 28,256) in 2024. The lowest positivity rate was observed in the summer of 2004 at 13.61% (152 of 1,117), while the highest was 39.73% (2,905 of 7,312) in the spring of 2021 (Figure 1).

**Figure 2:**
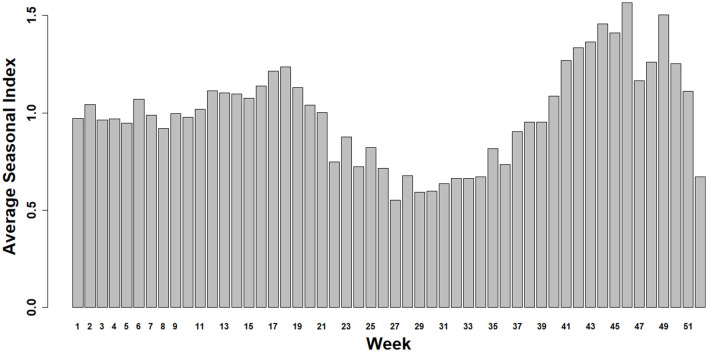
Average Seasonal Index for Influenza A virus from 2004 to 2024. The winter season is approximately represented by the bars between weeks 48 to 52 and 1 to 9, the spring season from weeks 10 to 22, the summer from 23 to 35, and the fall from 36 to 47.

The Average Seasonal Index was used to assess IAV seasonality from 2004 to 2024 (Figure 2). Subsequently, a bi-seasonal pattern of IAV detection emerged, showing increased detections during spring (March–May) and fall (September–November) (Figure 2).

**Figure 3:**
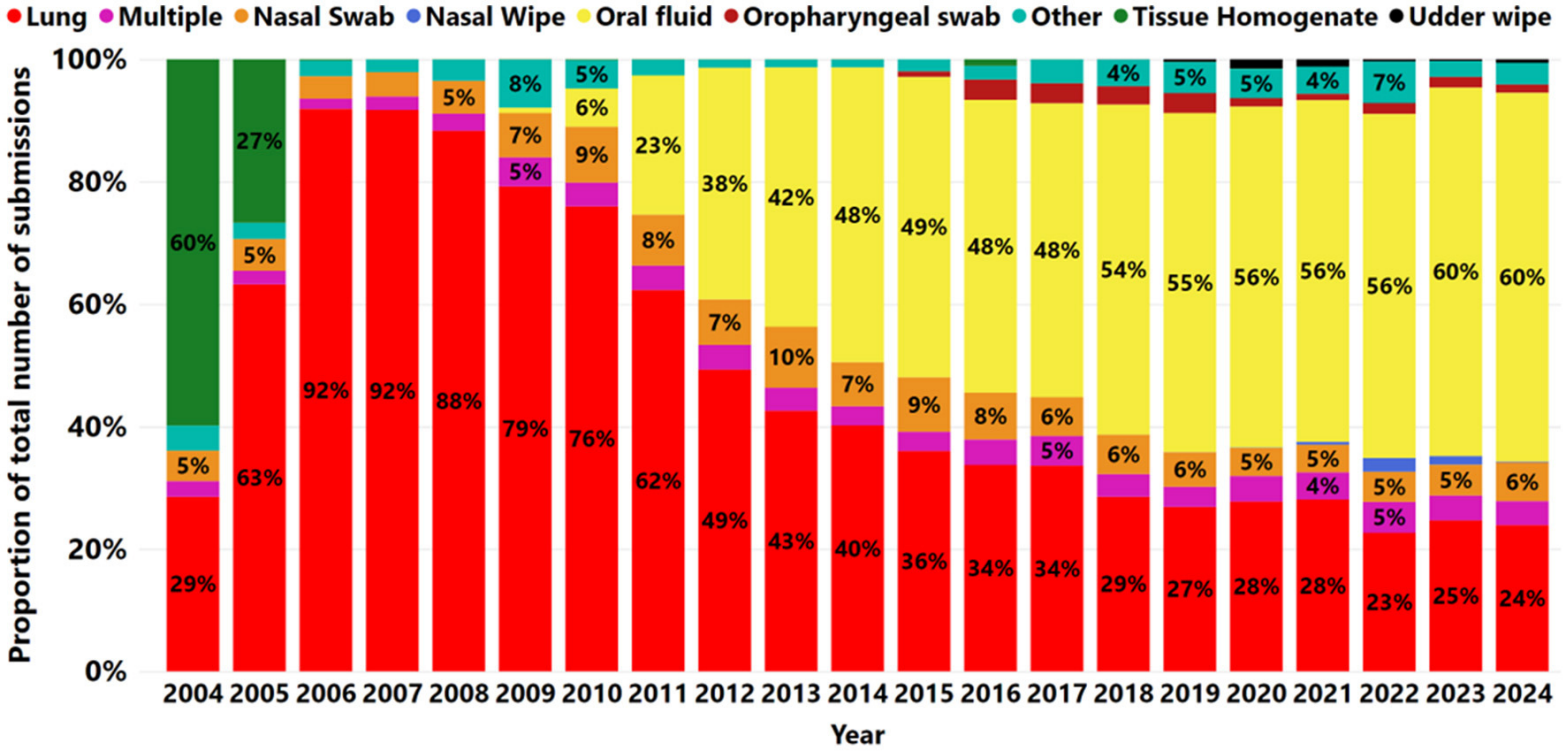
The proportion of specimens submitted for IAV RNA by RT-rtPCR from 2004 to 2024. Each year is illustrated by a bar. Different colors indicate different specimens tested for IAV by RT-rtPCR and the labeled numbers of a given specimen within a year.

Out of a total of 372,659 cases received, seven specimens represented 96.42% of the total: oral fluid 44.1% (164,510), lung 38.7% (144,061), nasal swab 6.2% (23,214), tissue homogenate 1.5% (5,548), oropharyngeal swab 1.4% (5,271), nasal wipe 0.34% (1,251), and udder wipe 0.32% (1,175) (Figure 3). In addition, multiple specimens comprised 3.8% (14,278) of the submissions. Furthermore, less common specimens, such as oral and bronchoalveolar lavage and tracheal swabs, were classified as “other” at 3.58% (13,351).

The proportion of samples submitted for testing using only oral fluid to detect IAV increased from 0.9% (60 of 6,742) in 2009 to 60.2% (17,025 of 28,256) in 2024. After 2013, oral fluid became the most frequently used specimen for IAV RT-rtPCR testing (Figure 3). The proportion of nasal swabs fluctuated between 4% and 6% from 2007 to 2024, with peaks observed in 2010 (9%) and 2013 (10%). A slight decline from 6% to 5% was observed between 2013 and 2017, followed by a more stable trend from 2017 to 2024 (Figure 3).

**Figure 4:**
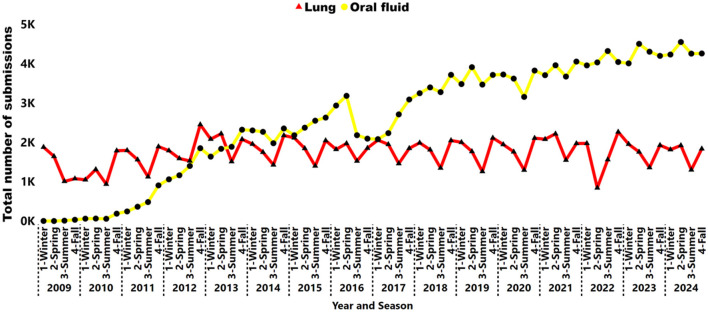
Number of lung samples (triangle red line) and oral fluid (circle yellow line) submissions tested for IAV by RT-rtPCR from 2009 to 2024. Each year is represented by season: 1, Winter; 2, Spring; 3, Summer; 4, Fall.

There was a decrease in the proportion of lung submissions from 92% (9,158 of 9,967) in 2006 to 24% (6,748 of 28,256) in 2024 (Figure 3). Nevertheless, the number of lungs remained steady from 2012 to 2024, averaging 7,240 cases per year over the last 13 years (Figure 4). A peak in lung submissions can consistently be detected during the winter months (Figure 4). The number of oral fluid submissions surpassed lung tissue submissions in 2013, suggesting increased surveillance over time within the swine population. Additionally, the tissue-lung specimen submissions showed consistent peaks in the fall season, from September to November, indicating that this specimen better reflects fall seasonality than other specimens (Figure 4).

Of the 372,659 submissions tested, the wean-to-market age category accounted for 43% (159,200), while the adult/sow farm represented 15% (54,118), and unknown age accounted for 43% (159,310). Over time, the proportion of submissions in the wean-to-market category has increased each year, rising from 39% (2,190 out of 5,622) in 2004 to 47% (13,240 out of 28,256) in 2024. Similarly, adult/sow farm submissions have increased their share, growing from 5% (339 out of 6,932) in 2004 to 21% (5,895 out of 28,256) in 2024. These changes suggest that while wean-to-market submissions have consistently represented the largest reported age category, adult/sow farm submissions have shown the highest proportional increase over this period, according to the participant VDLs database.

**Figure 5:**
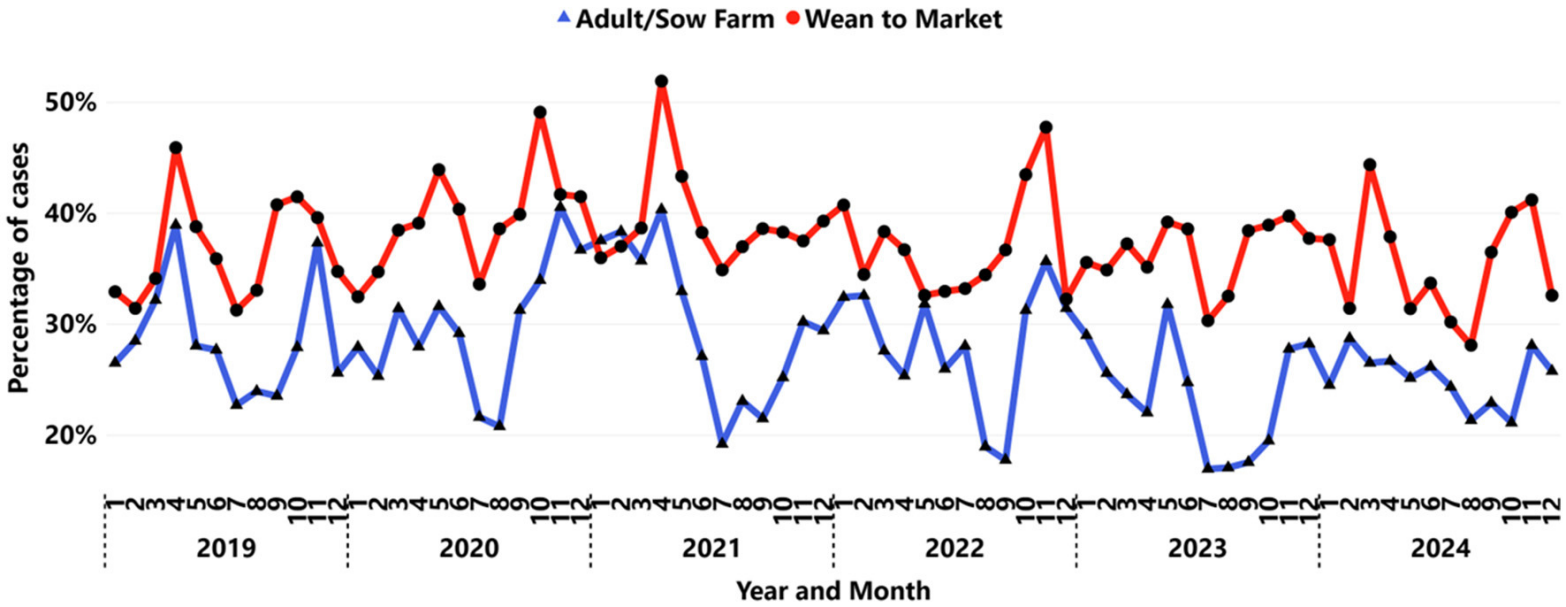
Monthly IAV positivity by RT-rtPCR presented by age category: adult/sow farm (triangle blue line), wean-to-market (circle red line).

The increase in submissions across both age categories also revealed notable differences in IAV detection patterns. Submissions from wean-to-market samples displayed a higher positivity rate (34.4%) than those from the adult/sow farm age category (26.9%), showing a significant (*P* < 0.001) statistical difference at the 0.05 level. Additionally, a bi-seasonal trend in detection, based on IAV peaks, typically occurred twice during the calendar year, with most observations in the spring and fall (Figure 5). Between September and October, the divergence in positivity rates for wean-to-market and adult/sow farms reached its peak, with an average difference of 12%, rising from 22% for sow farms to 34% for wean-to-market. Moreover, an earlier increase in IAV detection was noted in samples collected from wean-to-market approximately a month before the rise in detection in samples from adult/sow farms.

### 3.2 IAV subtypes RT-rtPCR results

**Figure 6:**
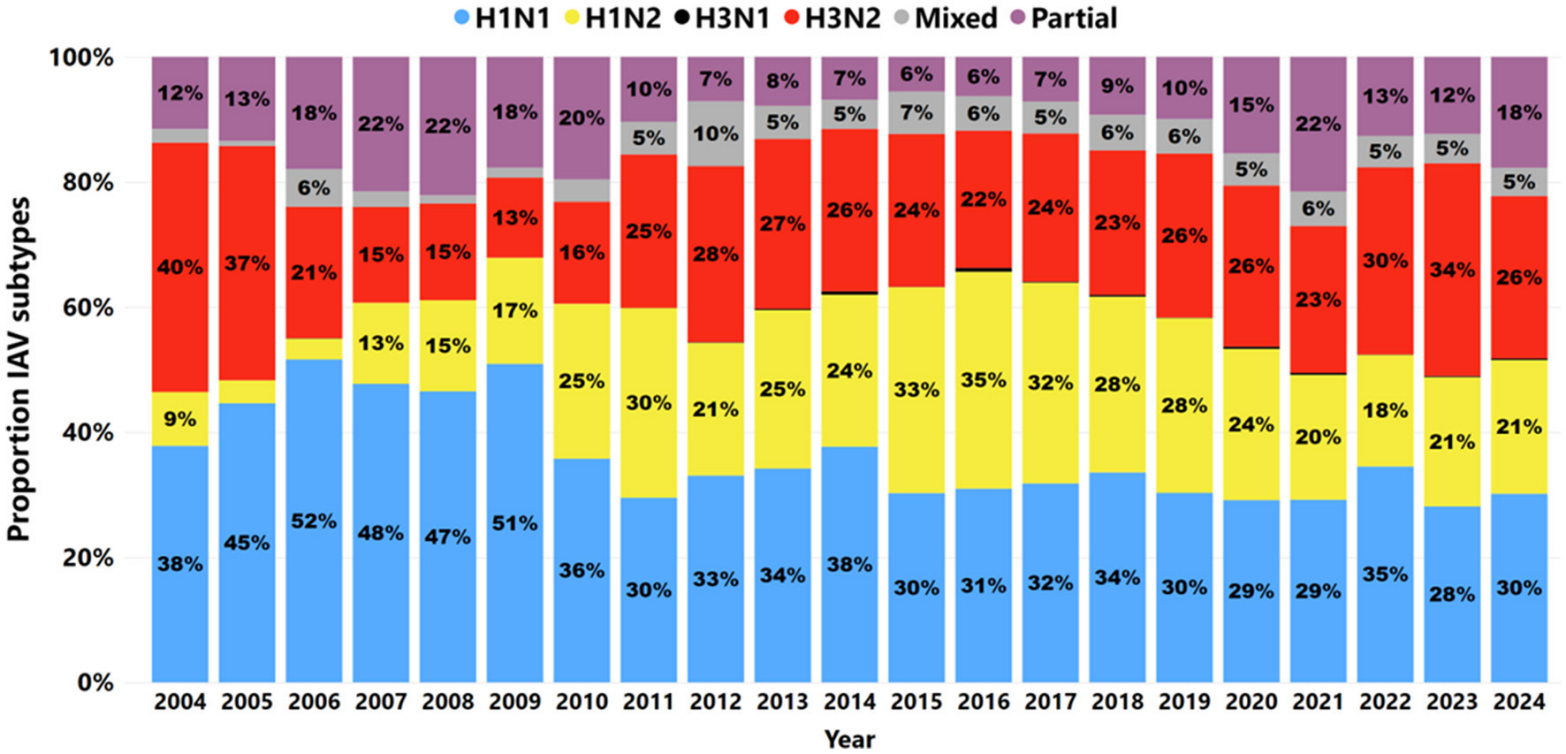
The proportion of IAV subtypes detected by RT-rtPCR from 2004 to 2024. H1N1 subtypes are denoted by blue; H1N2 subtypes are denoted by yellow; H3N1 subtypes are denoted by black; H3N2 is denoted by red; mixed subtypes are denoted by gray; partial subtypes are denoted by purple.

A total of 139,036 samples were tested for IAV subtypes, with the number increasing over time from 749 samples in 2004 to 7,456 in 2023 and then 5,586 in 2024, indicating a decrease in samples submitted. In 14.78% (20,546 of 139,036) of cases, no targeted IAV HA or NA subtypes were detected by RT-rtPCR. From 2004 to 2024, 85.22% (118,490 of 139,036) of samples tested revealed at least one IAV HA or NA-targeted subtype. The number of IAV samples subtyped rose from 685 cases in 2004 to 5,033 in 2024, with the average number of cases tested increasing from 57 per month in 2004 to 419 per month in 2024 (Figure 6). The H1N1 subtype was the most frequently detected, accounting for 33.13% (39,252 of 118,490), followed by H3N2 at 25.48% (30,186 of 118,490), H1N2 at 24.32% (28,822 of 118,490), and H3N1 at 0.2% (241 of 118,490). Partial subtype detection occurred in 11.2% (13,601 of 118,490) of the samples tested.

**Figure 7:**
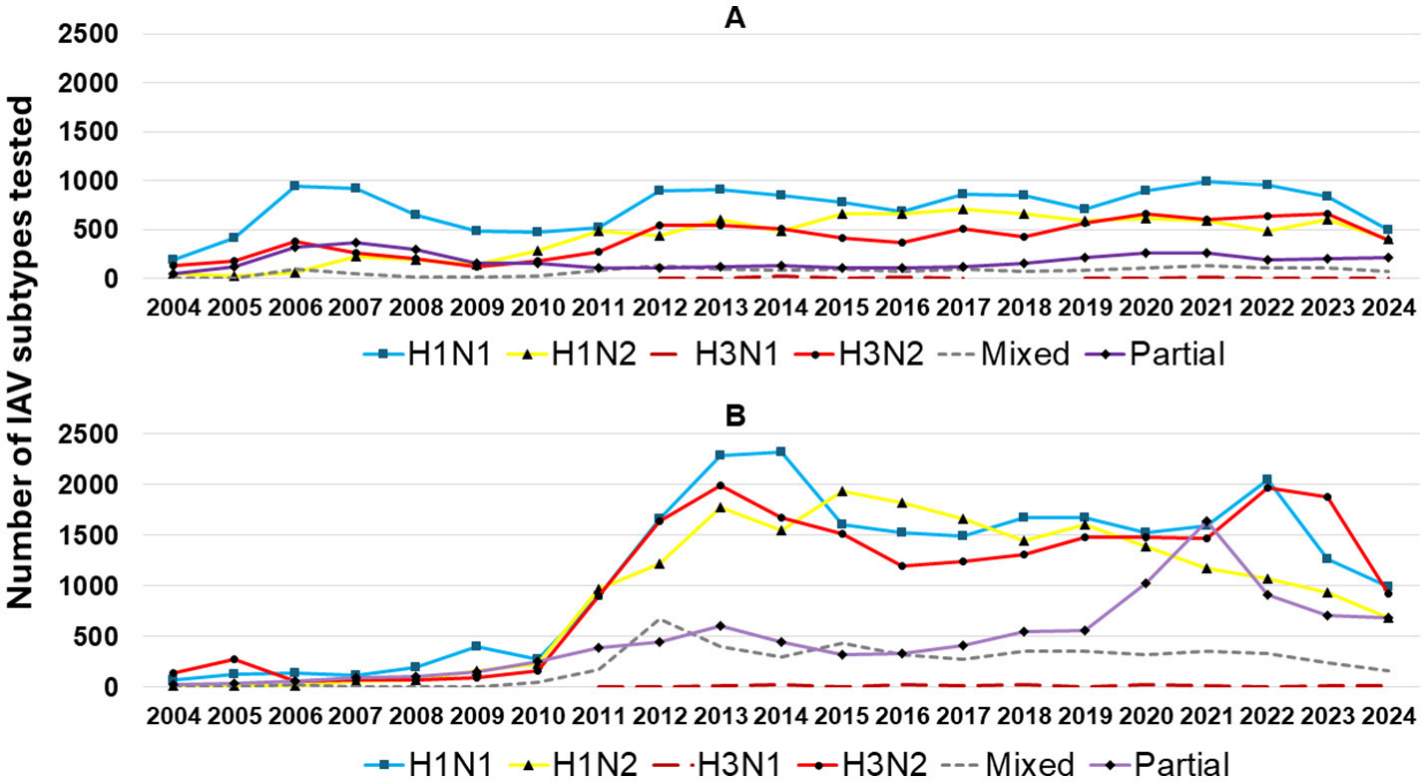
IAV subtypes detected in lung specimens **(A)** and other sample types **(B)** by RT-rtPCR from 2004 to 2024 are represented as follows: H1N1 subtype (blue squares), H1N2 subtype (yellow triangles), H3N1 subtype (dark red dashed lines), H3N2 subtype (red circles), mixed subtypes (gray dashed lines), and partial subtypes (purple diamonds).

From 2004 to 2009, H1N1 was a frequently detected IAV subtype, with an average detection rate of 47% during this period. After 2009, H1N1 detection decreased, while H1N2 and H3N2 became more prominent in recent years. The increase in H1N2 and the consistent presence of mixed subtypes suggest evolving influenza dynamics in swine populations. This dynamic of detection was also reflected in specimens; however, in lung samples (Figure 7A), the H1N1 subtype remained the most frequently detected, with stable detection over time. H1N2 and H3N2 became more frequently detected in the lungs in 2010 than in previous years. Mixed subtypes had lower but relatively consistent detection rates, while partial subtypes varied minimally from 2012 to 2017.

Mixed subtyping detection was observed in 5.4% (6,388 out of 118,490) of the samples tested. These detections were most common in oral fluid, accounting for 61.1% (3,906 of 6,388), followed by lung samples at 25% (1,599 of 6,388). Other detection sites included nasal swabs at 6% (378 of 6,388), bronchoalveolar lavage at 0.8% (48 of 6,388), nasal wipes at 0.6% (38 of 6,388), udder wipes at 0.5% (32 of 6,388), bronchial swabs at 0.4% (23 of 6,388), and oropharyngeal swabs at 0.1% (9 of 6,388). Supplementary specimens with fewer submissions, such as environmental samples and tracheal swabs, were grouped as “other” at 5.6% (355 of 6,388). Notably, mixed subtype detections were found in 38.4% (2,450 of 6,388) of individual sample types, including lung, nasal swab, and bronchoalveolar lavage (Table 1). These results underscore a notable prevalence of mixed subtype infections within individual specimens, with combinations H1H3N1N2, H1H3N2, and H1N1N2 being the most frequent across various sample types (Table 1).

**Table 1:** Proportion and number of mixed IAV subtypes detected in samples.

**Mixed subtypes**	**Percentage %, number of samples (n)**							
	**Oral fluids**	**Lung samples**	**Nasal swab**	**Bronchoalveolar lavage**	**Nasal wipes**	**Udder wipes**	**Bronchial swab**	**Oropharyngeal swab**
H1H3N1	3.6 (139)	6.3 (100)	3.7 (14)	8.3 (4)	–	3.1 (1)	–	11.1 (1)
H1H3N2	22.5 (878)	24.6 (393)	29.1 (110)	27.1 (13)	68.4 (26)	15.6 (5)	39.1 (9)	44.4 (4)
H1N1N2	33.2 (1,295)	39.0 (623)	36.0 (136)	35.4 (17)	18.4 (7)	46.9 (15)	30.4 (7)	22.2 (2)
H3N1N2	2.5 (106)	1.6 (25)	2.9 (11)	–	5.3 (2)	12.5 (4)	4.43 (1)	11.1 (1)
H1H3N1N2	38.1 (1,488)	28.6 (458)	28.3 (107)	29.2 (14)	7.9 (3)	21.9 (7)	26.1 (6)	11.1 (1)
Total	100 (3,906)	100 (1,599)	100 (378)	100 (48)	100 (38)	100 (32)	100 (23)	100 (9)

When excluding lung samples and retaining the remaining specimens in the analysis (Figure 7B), a sharp increase in overall IAV subtype detection was observed after 2010, likely driven mainly by increased oral fluid testing. There was a dynamic detection scenario, with H1N1, H1N2, and H3N2 being interchangeably detected over time. Mixed subtypes in non-lung tissues declined after 2012, and partial subtypes exhibited a marked rise from 2019 to 2021, suggesting an issue with the IAV RT-rtPCR subtype test based on increasing genetic diversity and a subtyping PCR that no longer detected all of the subtypes in circulation (Figure 7B).

### 3.3 IAV RT-PCR detection forecasting and monitoring

Five models were fitted to forecast expected levels of IAV detection by RT-rtPCR for 2024, using the previous 4 years (i.e., 2020 to 2023) as a baseline, following an 80:20 split of training data to testing data. After fitting the models, two models, SARIMA and neural network models, showed no significant autocorrelation (*P*-value ≥ 0.05) in their residuals, while the dynamic regression model exhibited weak autocorrelation (see Table 2). The dynamic regression model and neural network model, which had the lowest RMSE among models without or with weak correlation, were combined into a single model. For 2023, this combined model demonstrated superior performance, achieving the lowest RMSE and enhancing the overall skill score, where a higher skill score indicates better model performance. For 2024, the combined model had an RMSE of 4.76 and a skill score of 0.18, reflecting improved forecasting accuracy compared to the other models. However, the higher skill score from the combined model did not signify better performance than the individual models when forecasting for 2024, suggesting that the individual models could also have been utilized in 2024.

**Table 2:** Forecasting assessment: Ljung–Box test, RMSE, MAE, MAPE, and skill score.

**Model**	**Ljung–Box test 2023**		**Ljung–Box test 2024**		**RMSE**	**MAE**	**MAPE**	**RMSE**	**MAE**	**MAPE**	**Skill score**	
	**Stat**	***P*** **-value**	**Stat**	*P* **-value**	**2023**			**2024**			**2023**	**2024**
Dynamic regression	5.29	0.87	21.6	0.02	4.68	3.65	12.9	4.51	3.75	13.1	0.20	0.23
Cyclic	123	< 0.0001	84.3	< 0.0001	4.59	3.69	13.1	4.41	3.47	11.8	0.21	0.24
Neural network	3.7	0.96	11.8	0.28	4.58	3.6	13	5.33	4.58	16.1	0.23	0.08
Prophet	91.6	< 0.0001	77.4	< 0.0001	3.64	2.67	9.03	4.57	3.46	8.74	0.37	0.21
SARIMA	18.9	0.04	8.13	0.61	5.82	4.67	14.9	7.21	6.29	24.2	0.003	0.23
Combination^a^	–	–	–	–	4.35	3.42	12.3	4.76	4.07	14.3	0.26	0.18

^a^Combination of the dynamic regression and neural network models.

**Figure 8:**
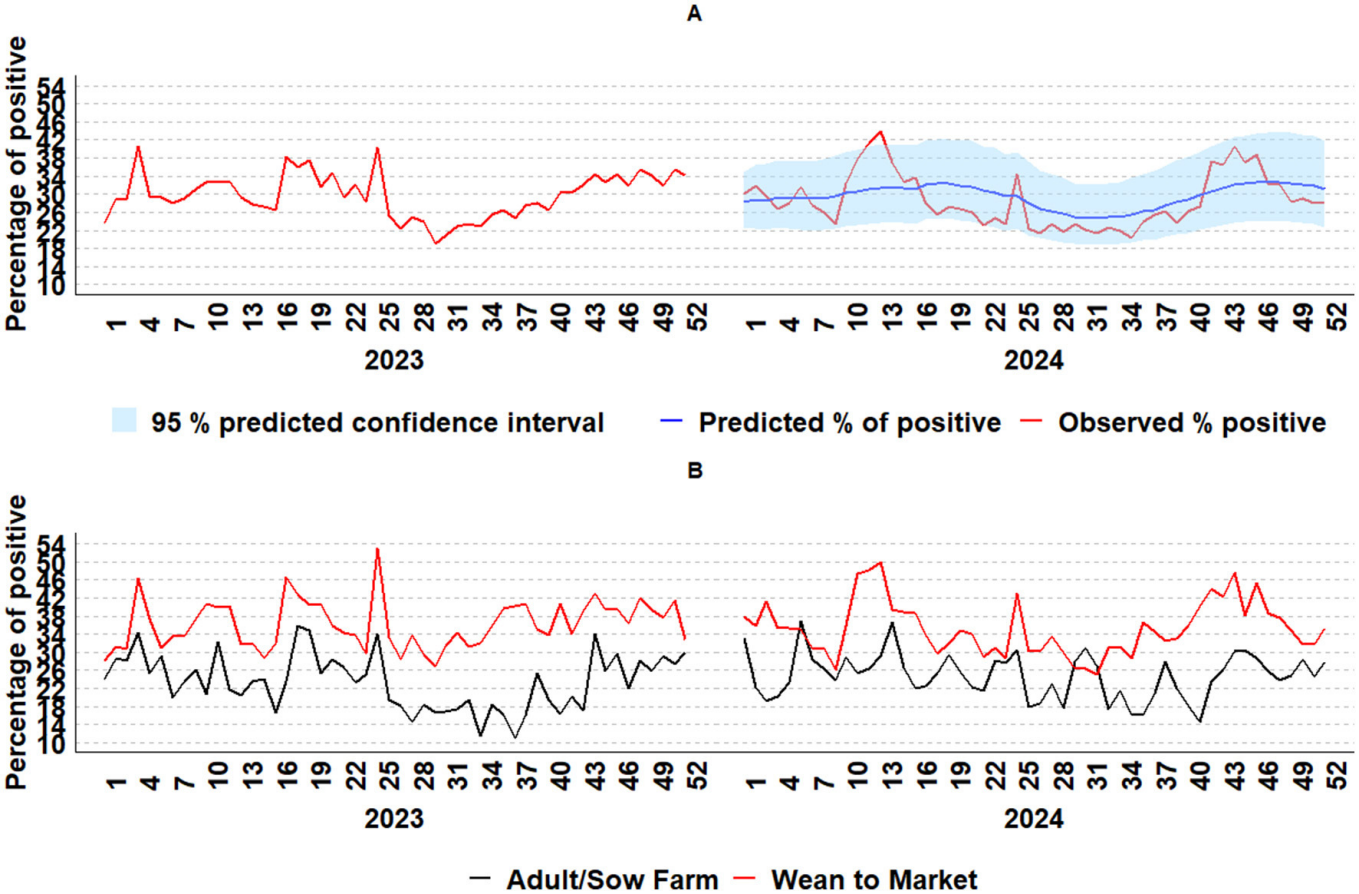
IAV weekly percentage of positive data monitoring from 2023 to 2024 by RT-rtPCR using a combination model with dynamic regression and a neural network model **(A)**; IAV weekly percentage of positive by age category **(B)**: adult/sow farm (black line) and wean-to-market (red line).

The generated combination model, which includes dynamic regression and neural network models, provided the predicted weekly IAV detection by RT-rtPCR values for 2024. The predicted 2024 weekly RT-rtPCR detection and its 95% prediction interval closely aligned with the observed data, with most data points falling within the 95% prediction interval (Figure 8A). For 2024, some deviations above the 95% prediction interval were observed in the wean-to-market category in week 12, with a 48% IAV positivity, and in week 13, with a positivity increase of 50% (Figure 8B). In 2023, occasional deviations above the 95% prediction interval were observed in the wean-to-market category in week 4, with a 46% IAV positivity, and in week 25, when positivity increased to 53%. The average positivity for the wean-to-market category in 2023 was 36.2%. Additionally, there was an increase of 14% (from 39% to 54%) in the proportion of lung sample submissions from week 3 to week 4 and an increase of 12% (from 32% to 44%) from week 24 to week 25, indicating higher IAV detection and more tissue diagnosis submissions, which potentially represented more animals affected during these weeks. Furthermore, deviations below the 95% prediction interval were observed, particularly in the adult/sow farm category, with IAV positivity of 16.9% in week 16, 15.4% in week 27, and 11.6% and 10.6% in weeks 34 and 37, respectively. The average positivity for the adult/sow farm category in 2023 was 23.9%, highlighting examples where the model was less effective at capturing unexpected declines in positive cases. Additionally, in 2022, there was an increase above the 95% prediction interval that started in October, from week 41 to week 45, aligned with an increase in the detection of IAV from both the adult/sow farm and the wean-to-market category.

A prediction analysis and model performance from 2018 to 2024 are presented in Table 3. Dynamic regression consistently showed lower RMSE and MAPE values in 2018, 2019, 2021, 2022, and 2024. However, in 2023, the Prophet model outperformed the others with the lowest RMSE and MAPE, while in 2020, the Cyclic Regression model achieved the best results for these metrics. Notably, the assumptions were met for the dynamic regression, neural network, and SARIMA models, except for SARIMA in 2019 and 2023. This indicates the changing complexity of forecasting each year. Overall, only the dynamic regression and neural network models did not consistently show autocorrelation of the residuals from 2018 to 2023.

**Table 3:** Forecasting model performance assessment from 2018 to 2024.

**Models**	**2018**			**2019**			**2020**			**2021**			**2022**			**2023**			**2024**		
	**RMSE**	**MAE**	**MAPE**	**RMSE**	**MAE**	**MAPE**	**RMSE**	**MAE**	**MAPE**	**RMSE**	**MAE**	**MAPE**	**RMSE**	**MAE**	**MAPE**	**RMSE**	**MAE**	**MAPE**	**RMSE**	**MAE**	**MAPE**
Dynamic regression	3.3	2.5	7.8	2.8	2.1	7.1	4.0	3.0	8.8	4.4	3.3	9.8	4.6	3.8	12.5	4.7	3.6	12.9	4.5	3.7	13.1
Cyclic regression	3.6	2.8	8.6	3.6	2.9	9.8	3.9	3.0	9.4	4.6	3.5	10.2	4.8	4.0	13.1	4.6	3.7	13.1	4.4	3.4	11.8
Neural network	3.9	3.3	10.3	4.2	3.3	11.5	4.2	3.2	9.7	5.1	3.7	11.0	6.7	5.5	18.0	4.6	3.6	13.0	5.3	4.6	16.1
Prophet model	4.8	3.9	12.3	3.2	2.6	8.1	5.1	3.9	11.2	7.4	6.2	20.0	5.1	3.9	11.8	3.6	2.6	9.0	4.6	3.5	8.7
SARIMA	5.6	4.2	12.9	5.5	4.5	13.5	4.8	3.6	10.5	8.8	7.4	24.0	7.0	5.3	16.6	5.8	4.7	14.9	7.2	6.3	24.2

## 4 Discussion

This study described the macroepidemiological aspects of IAV RNA detection through RT-rtPCR and its positivity distribution by age category and specimen. It highlights the importance of monitoring IAV using a standardized method to enable timely surveillance in the field. The primary aim was to present significant macroepidemiological aspects of IAV detection via RT-rtPCR, utilizing data from six NAHLN-accredited VDLs in the US, representing 344,301 submissions from 2004 to 2024. The IAV submission test and testing results from the participating VDLs in this project continue to be reported, and the findings are publicly available on the project website (SDRS, https://fieldepi.org/sdrs/). The secondary aim was to assess the presence of seasonality in the data and implement a weekly IAV monitoring system that analyzed the SDRS historical database for IAV detection, forecasted the expected results for the upcoming year, and provided predictions for weekly expected IAV detection results for the following year.

The change in the proportion of specimens submitted for IAV detection reflects evolving diagnostic preferences and potentially improved field sampling methods (Figure 3). The increasing proportion of oral fluid submissions—currently the predominant specimen type—suggests a growing reliance on this less invasive and more easily collected sample, which may enhance surveillance efforts. A similar finding was observed in a previous study (31), which reported increased use of oral fluids since 2011 for PRRSV and enteric coronavirus testing, contributing to the rise in submissions for herd monitoring purposes (32). Notably, after the validation of oral fluid samples for PRRSV and PCV2 monitoring and surveillance, the US swine industry increasingly adopted this sample type (59). Additionally, oral fluid has been demonstrated as a suitable sample for both PRRSV and IAV surveillance (60), and its widespread adoption by swine veterinarians and producers is largely attributed to its cost efficiency (61).

While lung tissue submissions have decreased proportionally over time (Figure 3), their consistent annual use for IAV RT-rtPCR testing (Figure 4) indicates that lung samples remain a crucial specimen type for specific clinical diagnostic purposes, as they have long been the preferred sample for molecular testing via RT-rtPCR assays (62). Lungs are typically submitted as reference samples from pigs exhibiting clinical signs and are used to confirm IAV diagnosis (63). In addition, individual pig samples, such as nasal swabs, have historically served as the gold standard antemortem sample for IAV diagnostics (27). However, individual animal sampling is labor-intensive and incompatible with the demands of modern, large-scale swine production systems (61). These samples limit the selection of donor pigs and reduce the likelihood of virus detection, as not all pigs may be at the same stage of infection. This limitation can result in the overinterpretation of disease presence if samples are not taken from acutely affected, representative animals. In contrast, population-based samples (e.g., oral fluids) allow sampling from multiple animals and simultaneously increase the likelihood of capturing pigs at the peak of virus shedding and improve detection sensitivity (63).

A bi-seasonal pattern of IAV detection in swine samples (Figure 2), with increased detection in the spring and fall, reflects a distinct seasonal trend compared to other swine pathogens. For example, previous studies have reported a single peak for PRRSV and enteric coronavirus, with the highest detection typically occurring during the colder months of the year (32, 36). One study identified that the wean-to-market age group exhibited higher positivity rates than the adult/sow farm category, particularly during seasonal peaks, suggesting that this group may serve as a reservoir or amplifier for PRRSV transmission (64). Similarly, higher IAV detection rates were observed in the wean-to-market group, indicating that this age category may also serve as a significant reservoir for IAV during peak periods. Moreover, a study identified an association between the season of weaning and the timing of placement into wean-to-market sites where IAV and PRRSV were detected, showing an increased length of the growing period, potentially due to the negative impact of these viruses on health and growth performance during the growing phase (65). The observed differences in positivity rates between these age categories, particularly during the fall, suggest that surveillance efforts may benefit from being tailored to specific production phases in order to more effectively anticipate and mitigate outbreaks.

An increase in IAV submissions over time has been significantly influenced by the high number of oral fluid specimens (Figure 4), particularly from 2010 onward. The number of samples classified as unknown has decreased over the years, indicating improvements in the data capture process conducted by VDLs. Oral fluid samples were initially described for PRSSV detection in 2008 (66), began testing for IAV in participant VDLs in 2009, and surpassed lung samples in 2013. Oral fluids are effective for monitoring IAV detection in the swine population, requiring fewer specimens compared to individual samples (67), and are routinely used for surveillance, thereby effectively monitoring subclinical cases and enabling early detection (61). The oral fluid sample serves as a powerful tool for monitoring and surveillance. Collecting oral fluid samples geospatially distributed across six pens of 60 pigs each in a 1,200-finishing barn allows for the detection of specific pathogens at a prevalence lower than 1% (68).

Swine influenza contributes to chronic respiratory disease problems and the presentation of Porcine Respiratory Disease Complex, a syndrome resulting from co-infection with two or more respiratory pathogens such as PRRSV (69). Surprisingly, lung specimens showed a recurring pattern of increased submissions during the fall season over time (Figure 4). Furthermore, this consistent rise may suggest elevated clinical respiratory cases linked to pig mortality in the fall, thereby favoring the submission of lung samples, which are postmortem specimens useful for diagnosing clinical disease in conjunction with any present histopathological lesions. Additionally, a study in Ontario indicated that the fall months significantly impact the increasing number of weekly and monthly diagnostic and positive submissions for IAV infections within Ontario swine populations (70).

Submissions from the wean-to-market age category showed a higher positivity rate (34%) compared to those from the adult/sow farm category (26%). This higher rate in the wean-to-market phase may be attributed to factors such as high pig density, larger herd sizes, and the use of multiple sources in the nursery and grow-finish operations (71). A previous study reported recurrent increases in PRRSV detection in the wean-to-market group prior to increased detection in the adult/sow category, with increased PRRSV activity typically occurring in the second half of the year (36). Another recent study highlighted pig density as a key factor for mapping PRRSV risk in swine-dense areas of the US Midwest (72). Similarly, data from the current study show an increase in IAV detection in wean-to-market samples approximately 1 month before a rise in detection from breeding herd submissions (36).

In the context of the integrated and dynamic North American swine industry, millions of pigs are transported across regions within the US, potentially introducing and transmitting new pathogens to swine populations (63). Understanding the movement and spread of IAV across different geographical regions and between countries is crucial (73). In addition, a study showed that pigs were frequently transported to harvest facilities in vehicles that had not yet been cleaned or disinfected between loads and that transport vehicles were often shared by different pig owners—conditions that facilitate the spread of disease across large regions (74). Moreover, modern swine production systems often require pigs to be transported over long distances between multiple locations, each specialized in different growth stages. This movement further increases the risk of further spreading IAV among swine populations (73). This pattern of pig movements within the US highlights the complexity of swine management and represents a potential route for the long-distance spread of IAV within the US (75).

These findings highlight the critical importance of reinforcing farm-level biosecurity practices by producers and veterinarians to reduce the spread of respiratory diseases such as IAV (64). This dynamic scenario also underscores the need to improve surveillance and monitoring systems to effectively manage and mitigate IAV transmission.

The H1N1 subtype was the most frequently detected between 2010 and 2021 (Figure 6), followed by H1N2 and H3N2 as the second and third most commonly detected subtypes, respectively. Interestingly, partial detection of H1, H3, N1, and N2 subtypes ranged from 18% to 20% between 2006 and 2010, with a notable increase from 10% in 2011 to 21.0% in 2021 (Figure 6). These variations in partial subtype detection may be impacted by the lack of ability to determine the full virus subtype in some samples, the presence of low viral loads below the detection threshold of the assays (76), and the high reassortment rate and ongoing evolution of IAV (77, 78).

In addition, the increase in oral fluid submissions (Figure 4) may also have been an important factor contributing to these partial detections, considering that IAV subtyping RT-rtPCR is less sensitive when targeting the genetically variable HA and NA genes compared to the screening RT-rtPCR assay, which targets more conserved regions of the genome. The increase in partial subtype detection may indicate a need to readjust and update the primers and probes used for IAV subtyping by RT-rtPCR to ensure the inclusion of contemporary virus strains that have evolved since the PCR was developed. Monitoring circulating IAV subtypes is both necessary and essential for epidemiological surveillance, supporting veterinarians in establishing prevention and treatment measures on affected farms, and guiding vaccine development (79). Furthermore, mixed subtype detections in lung samples indicate co-infection with multiple IAVs within individual animals, suggesting the presence of multiple IAV subtypes in a single lung sample, and highlighting the importance of addressing IAV infections as a multi-strain dynamic.

Detection of mixed subtypes further highlights the complexity of IAV epidemiology, as co-detections and potential viral reassortments are becoming increasingly frequent (Figure 6). Thus, several factors may contribute to the mixed detection of IAV subtypes in swine by PCR, including true co-infection, primer mismatches, and the co-circulation of different viral lineages (80, 81). A recent study showed that not all samples with a cycle threshold (Ct value) below 30 could be successfully subtyped, a limitation potentially attributable to specimen quality or the design of the primers used in the subtype multiplex RT-rtPCR assay (30). In this study, 24.6% of mixed IAV subtype detections were identified in lung samples, indicating that a single pig can be simultaneously infected with two identified IAV viruses—an occurrence less commonly expected in population-based sample types.

The relatively frequent detection of H1N1, H1N2, and H3N2 subtypes in lung samples (Figure 7A) contrasts with the more variable detection patterns in non-lung samples (Figure 7B), where H3N2 has shown increased detection since 2020. The rise in mixed and partial subtypes in non-lung samples post-2019 may be related to the increased use of oral fluids, indicating co-detections from sampling performed on a group of animals. Moreover, the number of samples tested annually has increased, particularly after 2012, suggesting enhanced surveillance efforts.

Combining multiple forecasts derived from various forecasting methods is often more effective than relying on a single forecast, as it incorporates multiple drivers of the data-generating process and mitigates uncertainties associated with model form and parameter specification (51). Moreover, integrating diverse forecasting models enhances overall robustness (82). The combined forecasting model, which incorporates dynamic regression and neural network approaches, demonstrated superior performance in predicting IAV positivity trends for 2023. The improvement in RMSE and skill score indicates that combining different forecasting methods can enhance the accuracy of IAV surveillance models, with combination models achieving higher skill scores compared to individual models when capturing seasonal patterns and deviations for 2023. A deviation in week 25 of 2023 occurred during late spring and may relate to environmental factors such as temperature shifts, manure pumping, poor air quality within barns, and co-infections, prompting veterinarians to submit samples to diagnostic laboratories for IAV detection (18). Additionally, there was a 12% increase in the proportion of lung submissions (from 32% to 44%) from week 24 to week 25 of 2023, indicating increased IAV detection and more tissue diagnosis submissions, potentially representing a higher number of affected animals during these weeks. Moreover, in 2022, there was an increase above the predicted level in October for both the adult/sow farm category and wean-to-market, suggesting IAV activity coinciding with seasonal changes and manure pumping in many regions. Although not included in the scope of this study, it is important to note that the presence of other respiratory pathogens, such as PRRSV, *Mycoplasma hyopneumoniae*, and *Pasteurella* species, can also influence the severity of influenza cases in swine during this period, as PRRSV and these endemic bacteria may also exhibit increased activity in the fall.

This study has some limitations that need to be addressed. First, this is aggregated data from submissions with multiple purposes, including clinical samples and surveillance testing for IAV. Thus, because samples were submitted for various purposes, inferences drawn from aggregated results about prevalence, incidence, diagnostic sensitivity, and specificity cannot be made with statistical confidence. The shared data do not provide site-specific identification, resulting in an inability to track recurring sampling or determine how long IAV has been detected at a given site. Additionally, when using VDL data, it is essential to recognize that the test results are based on samples submitted specifically for diagnostic purposes and, therefore, do not represent the IAV prevalence or incidence. IAV subtype data is based on predefined sets of primers and probes, which may limit broader conclusions regarding IAV genetic diversity that could be captured by sequencing techniques like Sanger and next-generation sequencing. Nevertheless, there may be subpopulations of pigs in the US utilizing other VDLs for IAV testing that are not included in this work, potentially creating regions or subpopulations in the US that may be underrepresented in this study.

## 5 Conclusion

This study describes the macroepidemiological aspects of IAV RNA detection and its distribution according to age category, specimen, and seasonal detection trends from the six major VDLs in the US participating in SDRS from 2004 to 2024. It also describes the IAV subtype diversity over time, with frequent detections of IAV mixed subtypes in lung tissues, suggesting co-infection of individuals with multiple IAV subtypes. In addition, this study highlights the importance of the wean-to-market age category for IAV detection dynamics, with higher positivity rates compared to the adult/sow farm category. Overall, oral fluid and nasal swabs were the most frequent antemortem samples submitted for diagnostics, while lung tissue was the predominant postmortem sample, highlighting their importance in IAV detection and surveillance. In summary, data from this study suggest that IAV has a biseasonal pattern of detection from swine samples, and subtyping detection dynamics are constantly occurring over time. This study outlines the importance of monitoring the influenza virus and its subtypes in a standardized way, thus enabling timely surveillance and more effective decision-making based on the macroepidemiological information provided by this project in the US.

## Data Availability

The original contributions presented in the study are included in the article/supplementary material, further inquiries can be directed to the corresponding author.
